# Insights into epigenetics suggest a role of DNA methylation in regulating Chinese yam tuber shape

**DOI:** 10.1186/s12870-026-08438-5

**Published:** 2026-03-02

**Authors:** Jenny Riekötter, Toshiyuki Sakai, Akira Abe, Ryohei Terauchi, Janina Epping

**Affiliations:** 1 University of Münster, Institute of Plant Biology and Biotechnology, Münster, 48143 Germany; 2 Kyoto University, Crop Evolution Laboratory, Muko, Kyoto 617-0001 Japan; 3 Iwate Biotechnology Research Center, Kitakami, Iwate 024-0003 Japan

**Keywords:** Chinese yam, Tuber development, DNA methylation, Epigenetics

## Abstract

**Background:**

Plant organ shape is an important trait in terms of economic efficiency in agriculture. The club-like or spindle-shaped underground tubers of Chinese yam (*Dioscorea polystachya* Turcz.) impede mechanical harvest resulting in unprofitable cultivation.

**Results:**

We performed genome-wide DNA methylation sequencing of two closely related Chinese yam tuber shape variants F60 (long, thin) and F2000 (short, thick) to investigate potential epigenetic effects on tuber development and shaping. Analysis of differentially methylated regions (DMRs) led to the identification of 1,513 hyper- or hypomethylated regions in 763 DMR-associated genes of which the majority were detected in the CHG methylation context. Gene Ontology (GO) term analysis revealed enriched DMR-associated genes related to anatomical structure development, hormone response, and photomorphogenesis. DMRs in brassinosteroid (BR) pathway-related genes, in particular BR signaling genes, were detected. We further identified hyperDMRs and hypoDMRs in putative circadian rhythm-related genes such as *DpVOZ1*, *DpPIE1* and *DpSPA1* as well as several putative organ shape-related genes (*DpIQD13, DpTCPs*).

**Conclusion:**

A potential network consisting of components of the BR signaling pathway, and circadian clock as well as microtubule-associated proteins was suggested to coordinate tuberization and affect tuber shape of Chinese yam.

**Supplementary Information:**

The online version contains supplementary material available at 10.1186/s12870-026-08438-5.

## Background

The effects of epigenetics on plant development have been intensively studied in the past decades [1–3]. Epigenetic regulators include DNA methylation, histone modifications, non-coding RNAs and nucleosome positioning, which, in contrast to genetic changes, can be reversible and affect gene expression without genomic sequence alterations [4–6]. In plants, DNA methylation of cytosine (5-methylcytosine) occurs in the three contexts: CG, CHG and CHH (where H represents C, T or A) [3, 5].

DNA methylation has been reported to affect genome stability, gene expression and transposon silencing, which can be inherited (genetic imprinting), as well as temporarily dynamically changed depending on the plant developmental stage and/or as a response to certain stimuli e.g. environmental stresses [5, 7–10]. In addition, investigations of DNA methylation profiles have revealed a tissue-specific methylation pattern in *Arabidopsis* [11, 12]. Typically, reduced DNA methylation levels around the transcription start site (TSS) and the transcription termination site (TTS) are observed in plants [13]. While hypermethylation in the promoter as well as in the transcriptional termination region is often associated with gene suppression or silencing, hypermethylation within gene bodies may cause the activation of gene expression [14–19]. Furthermore, DNA methylation within exons has been reported to affect alternative splicing events, thereby regulating transcriptomic and proteomic diversity [20].

The effect of DNA methylation on plant developmental processes such as dormancy release, seed germination, fruit development, fruit ripening, and grain filling has been investigated in previous studies [21–26]. Interestingly, a connection between DNA methylation and organ size was shown in chickpea, where comparison of methylome and transcriptome of two cultivars differing in seed size led to the identification of differentially methylated and expressed genes related to cell cycle and grain filling indicating a DNA methylation-mediated regulation of seed development [27]. Moreover, reduced methylation in the *fvefdm1* woodland strawberry mutant caused a severe phenotype with reduced leaf, flower and fruit size [24]. The *fvefdm1* gene shares high similarity to *Factor of DNA Methylation1* (*FDM1*) from *Arabidopsis*, encoding a component of the RdDM pathway [24].

To date, no research on DNA methylation in Chinese yam (*Dioscorea polystachya* Turcz.) has been reported and only limited information on epigenetic effects in plant tuber shaping is available [28]. Chinese yam is a polyploid, monocotyledonous plant species, that produces nutritious underground tubers that derive from the hypocotyl [29–31]. The vegetative propagation by seed tubers or aerial tubers, known as bulbils, is very common and results in clonal offspring [32]. This crop is native to Asian countries like China, Korea, Kuril Island and Taiwan, where it has been grown for thousands of years, while being fairly unknown in western countries [32–36]. One major reason is the labor-intensive harvest that relies on manual digging due to the club-like tuber shape that impedes the use of harvesting machines [34, 37]. As the tubers grow deep in the soil (up to 1 m) and get thick at the distal end while remaining thin at the head region near the soil surface, pulling out of the soil would result in breakage of the fragile tubers and thus in harvest loss [34, 37, 38]. Generally, plant organ shape is regarded as an important agronomic trait that has been optimized in modern crops [39]. Therefore, improving the tuber shape towards more harvestable organs is a critical aspect for the profitable cultivation of Chinese yam [34]. For this purpose, understanding the molecular process of Chinese yam tuber shaping is crucial to optimize this species towards rounder tubers with specifically shorter head regions [34, 40]. Variant F2000 was derived from F60 by selecting thicker and shorter tubers over several decades using the asexual propagation method [40]. Thus, this inherited organ shape of the two variants might not necessarily be a result of genetic variation, but rather be affected by epigenetic modifications.

Hence, we investigated the genome of F60 and F2000 for differences in DNA methylation pattern by whole-genome bisulfite sequencing. We analyzed differentially methylated regions around and within genes in all three cytosine methylation contexts and identified genes related to organ shaping, flowering time control, and hormone biosynthesis and signaling. Our results provide new insights of the putative role of epigenetics in the molecular network regulating *D. polystachya* tuber development and shaping, which can be used as a base for further research.

## Methods

### Plant materials and DNA extraction

Plant material was obtained from clonally propagated Chinese yam tubers of variant F60 and F2000 that were collected and used in our previous published transcriptome study [40]. F2000 was derived from F60 via clonal propagation and selection for thicker and shorter tuber shape. Species identification was performed by DNA barcoding using the DNA barcode markers *mat*K and *rbc*L as described by Sun and colleagues [41]. Close genetic relationship of both variants were previously confirmed by ISAP marker PCR [40, 42]. In brief, tuber pieces of Chinese yam (*Dioscorea polystachya*) variant F60 (long, thin) and F2000 (short, thick) (provided by a local farmer from St. Claude de Diray, Loir-et-Cher, France) were planted in raised-bed gardens in April 2019 (Münster, Germany, 51°58′34.8″N 7°35′03.5″E). During enlargement stage (three months after sprouting), newly development tubers were harvested and divided into the upper head, middle and tip region. Tubers were freeze-dried and ground to fine powder. Genomic DNA was extracted from the middle tuber part using a modified protocol of Zeng and colleagues [43]: 1 mL ice-cold CTAB-free buffer and 6 µL precooled 2-mercaptoethanol were added to 30 mg tuber powder in a 2.0 mL tube and mixed followed by an incubation on ice for 10 min. After centrifugation (10,000 g, 4 °C, 10 min), supernatant was discarded. Pellet was resolved in 500 µL prewarmed (65 °C) CTAB buffer containing additional1% (w/v) PVP, 1% (w/v) SDS, 5 µL 2-mercaptoethanol and 10 µL Proteinase K (New England Biolabs, Ipswich, MA, USA) and incubated at 65 °C for 60 min. Tubes were cooled down to room temperature and one volume of chloroform:isoamyl alcohol (24:1 (v/v)) was added. After mixing by inversion for 1 min, samples were centrifuge (16,000 g, 15 min) and the upper aqueous phase was collected in a new 1.5 mL tube. The previous step was repeated until the aqueous layer became clear. Then, 1/2 volume 5 M NaCl was added and mixed via inversion. Subsequently, the sample was mixed with 2/3 volume ice-cold isopropanol and incubated at −20 °C overnight. DNA was pelletized by centrifugation (16,000* g*, 4 °C, 15 min) and supernatant was discarded. Pellet was washed twice with 1 mL 75% (v/v) ethanol each followed by a centrifugation step (16,000 g, 4 °C, 5 min). After drying the pellet at room temperature, 400 µL of high salt TE and 2 µL RNase A (ThermoFisher Scientific, Waltham, MA, USA) were added and incubated at 37 °C for 60 min. Sample was occasionally mixed until the pellet was completely dissolved. DNA was precipitated by adding 2 volumes of ice-cold 100% ethanol and incubating at −20 °C for 60 min. Sample was centrifuged (16,000* g*, 4 °C, 15 min) and pellet was washed twice in 75% (v/v) ethanol. The air-dried pellet was dissolved in 100 µL TE. Quantification of genomic DNA was performed using a Qubit 2.0 fluorometer, while sample integrity and purity was confirmed by agarose gel electrophoresis.

### Low input whole-genome bisulfite sequencing

Prepared DNA samples were sent to Novogene Company (Beijing, China) for low-input whole-genome bisulfite sequencing including the steps of bisulfite treatment and library construction. Negative control (λ genome DNA) was added into samples and DNA fragmentation into 200–400 bp fragments was performed using Covaris S220. The resulting ssDNA fragments were then bisulfite treated (Accel-NGS Methyl-Seq DNA Library Kit for Illumina, Swift) and methylation sequencing adapters ligation was conducted. Subsequently, size selection of the DNA fragments followed by PCR amplification steps were performed. Whole-genome bisulfite sequencing was performed on the NovaSeq 6000 (Illumina, San Diego, Ca, USA) platform. Libraries of three biological replicates per variant (F60 and F2000) were sequenced for 150 bp paired-end reads resulting in at least 18 G of raw data per sample.

### Methylation calling

For adapter clipping and removal of low-quality reads, PRINSEQ + + v1.2.4 [44] and Trimmomatic v0.40 [45] were used. Thereby, at least 10 bases of the left end of each read were clipped and quality trimming was performed using a 4-base sliding window and a quality average threshold of 15. Further, sequences shorter than 75 bp were removed. After quality control, high-quality filtered reads were aligned to the *D. polystachya* var. F60 genome (http://www.ebi.ac.uk/ena/browser/view/PRJEB82723) [46] using Bismark v0.23.1 [47] and Bowtie 2 with following parameters: –score_min L, 0, −0.6 and a maximum insert size of 700 bp between the paired-end alignments. Given the polyploid nature of yam, homologous regions can cause ambiguous alignments. Therefore, only uniquely mapped reads were retained for downstream analyses. Deduplication step was performed and methylation calling for the CG, CHG and CHH context was conducted ignoring the first 5 bp of the 5-primed end of each read using the corresponding methylation extractor option.

### Methylation level analysis and identification of differentially methylated regions

The R package methylKit v1.24.0 was used for methylation level and PCA analysis for each context [48]. For the identification of differentially methylated regions (DMRs), a minimum coverage threshold of 10 reads per cytosine was used followed by the sliding window approach with a window size of 100 bp and a step size of 100 bp. Methylation coverage 4 kb upstream of the transcription start site (TSS), within the gene body, and 4 kb downstream of the transcription termination site (TTS) were calculated and visualization of methylation levels was performed using deepTools v3.5.2 [49]. DMRs were specified as hypermethylated in F2000 with a methylation difference of ≥ 10% and q-value ≤ 0.01, whereas hypomethylated regions had a methylation difference of ≤ −10% and q-value ≤ 0.01. The R package genomation v1.30.0 was used to extract genomic annotation information [50].Venn diagrams were generated using InteractiVenn [51].

### Protein function annotation and enrichment analyses

Protein function annotation was assigned to protein sequences using the online tool Mercator4 v7.0 (https://www.plabipd.de/mercator_main.html, accessed on 28 January 2025) [52, 53]. Gene Ontology (GO) term and KEGG reaction identifier (KO) annotations were obtained using PANNZER2 (http://ekhidna2.biocenter.helsinki.fi/sanspanz/#, accessed on 11 March 2025) and the eggNOG mapper software (version emapper-2.1.12, accessed on 28 January 2025), respectively [54–57]. PANNZER2 annotation was run with following parameters: minimum query coverage of 0.4 or minimum subject coverage of 0.4, minimum alignment length of 50, and minimum sequence identity of 0.4. Annotation with eggNOG mapper was performed using default parameters. Genes were annotated with GO terms combining PANNZER2 and eggNOG results. To identify enriched GO terms of hyper- and hypomethylated genes, the R package GoSeq2 was used. KO annotations were assigned to KEGG pathways using the KEGG Mapper tool (https://www.genome.jp/kegg/mapper/) [58, 59].

### Interaction network of hyper- and hypomethylated regions

The prediction of a potential protein interaction network was performed using the online STRING platform (https://string-db.org/, accessed on 25 February 2025) [60]. Here, protein sequences were used as input and equivalent proteins in *Arabidopsis thaliana* were used for potential network analysis. Visualization of network plots was performed with Cytoscape v3.10.0 [61]. Homologous proteins without known network connection were excluded from the network plot.

### Integration of DNA methylation and gene expression analysis

We integrated the transcriptomic data of *D. polystachya* variants F60 and F2000 from our previous study with the DNA methylation data that were generation from the same sample material. Transcriptomic data of three biological replicates of F60 and F2000 head, middle and tip samples [40] (Accession number: PRJNA942579) were analyzed for differential gene expression as previously described [46]. Genes were considered as differentially expressed with a *p*-value ≤ 0.001 and FDR ≤ 0.05. Heatmap of DMR-associated differentially expressed genes was generated using TBtools [62].

## Results

### Distribution of DNA methylation in F60 and F2000 tubers

**Fig. 1:**
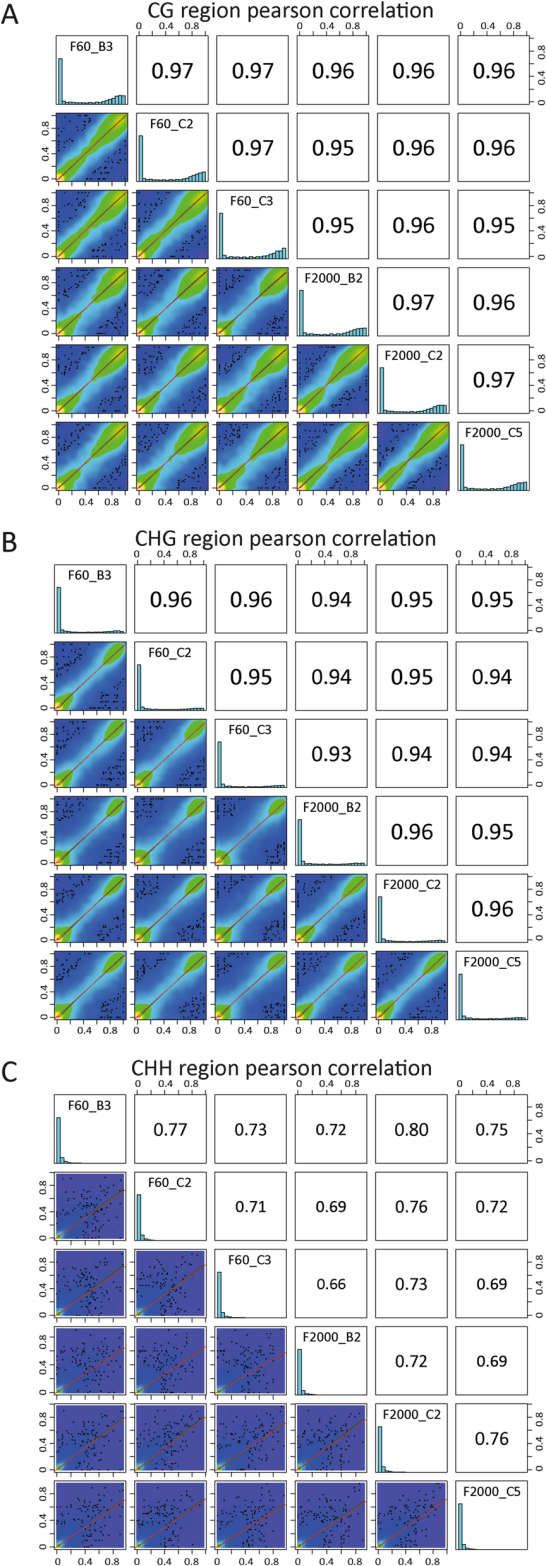
Scatter plots of genome-wide DNA methylation data between Chinese yam var. F60 and F2000 triplicates. **A**-**C** Resemblance of methylation values of base resolution between biological replicates were analyzed in the CG, CHG and CHH context given as Pearson’s correlation scores*.* Scatterplots contain a fitted linear regression line (red) and a polynomial regression line (green). Methylation level of each sample were plotted as histograms

To analyze the DNA methylation pattern of the two Chinese yam tuber shape variants F60 (long, thin) and F2000 (short, thick), bisulfite converted genomic DNA of F60 and F2000, each in biological triplicate, was sequenced resulting in 31.56 Gb for F60 and 30.63 Gb for F2000 (see Additional file 1). After filtering and quality check of the paired-end reads, an average of 59,657,959 for F60 and 58,608,303 clean reads for F2000 were obtained. Using lambda phage DNA, overall rate of BS conversion of unmethylated cytosine bases (from cytosine to thymine) was estimated. Here, the BS conversion rate was more than 99.7% for all biological replicates. Subsequently, clean reads were mapped against the recently published Chinese yam genome of variant F60 [46] resulting in an average mapping efficiency of 64.93% of uniquely mapped clean reads for F60 samples and 65.13% for F2000 samples before deduplication. In the next step, we analyzed the number of methylated cytosines in the CG, CHG and CHH context. A total of 18.66% in F60 and 17.91% in F2000 of the genomic cytosines were methylcytosines. Of all analyzed cytosines (C), the majority of methylated cytosines were observed in the CG context (8.14% in F60, 7.76% in F2000), whereas the least methylated nucleotides were detected in the CHH context (4.32% in F60, 4.12% in F2000). More than 72% of the analyzed cytosines in the CG context were methylated in both tuber shape variants (74.57% in F60, 73.07% in F2000), while almost half of the analyzed cytosines were methylated in the CHG context (> 47% for both variants). In contrast, only 5.63% and 5.4% of the cytosines in the CHH context were methylated in F60 and F2000, respectively. Pearson correlation analysis of the investigated samples revealed high correlation between the biological replicates of each variant in the CG (> 95%) and CHG (> 93%) context, however, lower resemblance between the biological replicates was observed in the CHH context (< 80%) (Fig. 1).

**Fig. 2:**
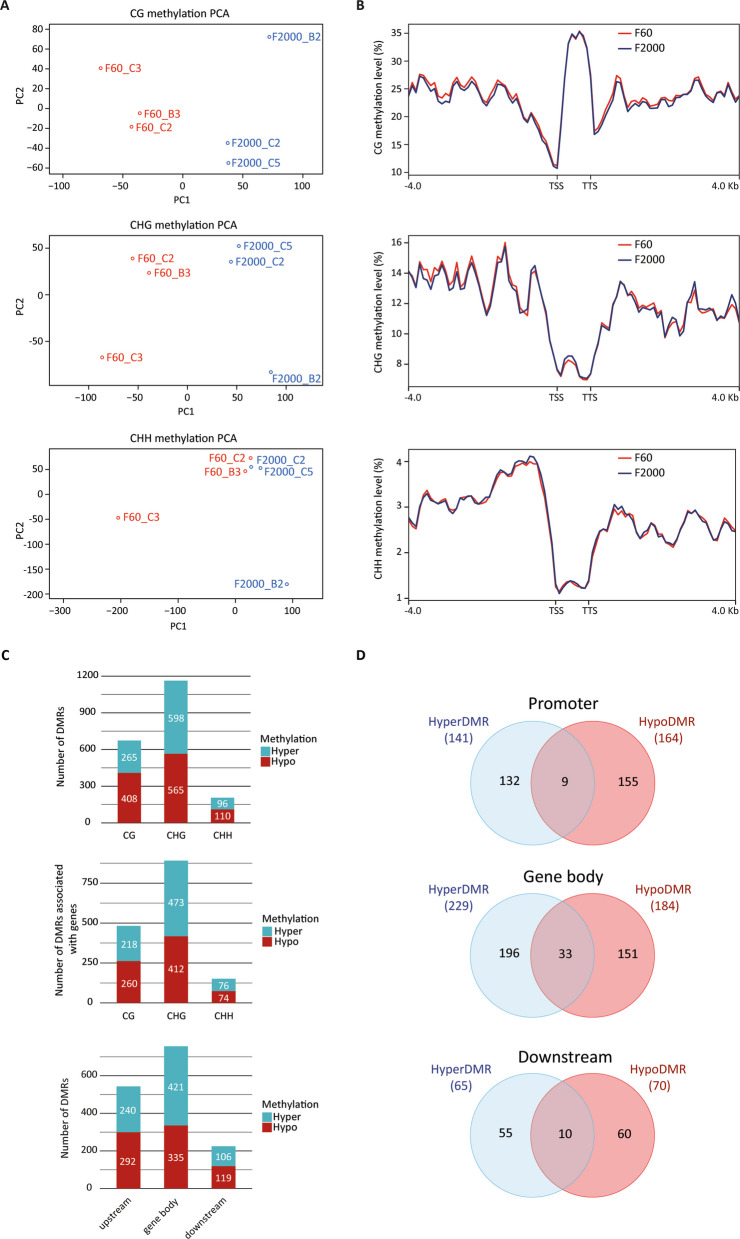
Whole-genome DNA methylation analysis of Chinese yam variant F2000 and F60. **A** Principal component 1 and principal component 2 analyses were performed for each sample. **B** Methylation level distribution across genetic features including 4 kb upstream of TSS, gene body, and 4 kb downstream of TTS in the three DNA methylation contexts CG, CHG, and CHH. Analyses were performed in biological triplicates for each variant. **C** Number of DMRs with higher methylation levels in F2000 (hypermethylated) and in F60 (hypomethylated). DMRs were investigated in the three DNA methylation contexts CG, CHG and CHH. Distribution of DMRs upstream in the putative promoter (4 kb of TSS), within the gene body, and down-stream in the putative terminator (4 kb of TTS) were analyzed in DMR-associated genes (q-value ≤ 0.01, methylation difference >|10%|). **D** Venn analysis of DMR-associated genes split in hyper- and hypomethylated promoters, gene bodies, and terminators. DMR, differentially methylated region; TSS, transcription start site; TTS, transcription termination site

Principle component analysis (PCA) confirmed these results, showing a clustering of the biological replicates of F60 and F2000 in the CG and CHG methylation context, respectively, indicating a similar methylation profile and thus a good consistency between the replicates, whereas two samples of F60 and F2000 clustered together in the CHH methylation context (Fig. 2A). We merged the data of the triplicates of each tuber shape variant and calculated the methylation level for each context. Using the tilling window approach methylated regions were analyzed instead of single cytosines. To investigate the methylation level profile around and within the gene body (from TSS to TTS), we extracted the coordinates of 4 kb upstream the TSS and 4 kb downstream the TTS, which were then divided into 100 bins. Methylation level dropped approaching the TSS followed by an increase in methylation level along the gene body and then decreased again towards the TTS in all three contexts, for the CG context, methylation was highest within the gene body, whereas opposite observations were made in the CHH and CHG context (Fig. 2B). Here, higher DNA methylation levels were detected upstream and downstream of the gene body. These observations were made in both tuber shape variants.

### Analysis of differentially methylated regions between Chinese yam tuber variants

To investigate differences in DNA methylation between the F2000 and F60 tuber shape variants, DMRs with a methylation difference of ≥|10%| and a q-value ≤ 0.01 based on Fisher’s exact test were considered for further downstream analysis. In total, 959 regions with higher methylation in F2000 (hypermethylated; hyperDMR) and 1,083 with higher methylation in F60 (hypomethylated; hypoDMR) were identified (Fig. 2C, see Additional file 2). Of these regions, 265, 598, and 96 were hypermethylated in the CG, CHG, and CHH context, respectively. Contrary to this, 408, 565, and 110 hypoDMRs were detected in the CG, CHG, and CHH context. We then investigated DMRs located within the gene body and its flanking regions (4 kb upstream and downstream). Of the 2,042 DMRs, 1,513 were located within or around 763 genes (DMR-associated genes). Majority of DMRs was located in the gene body (421 hyper- and 335 hypomethylated), followed by DMRs in the putative promoter region (240 hyper- and 292 hypomethylated), whereas lowest amount was detected downstream of the TTS (106 hyper- and 119 hypomethylated). Notably, DMR distribution varied in the three contexts. For the CHH context, DMRs were more abundant upstream of the TSS, while peak of CG and CHG methylation was detected in the gene body (see Additional file 2).

Next, we examined the intersections between hyperDMRs and hypoDMRs in the three genetic features by performing a Venn analysis. For this purpose, we extracted the gene identifier (gene IDs), as multiple DMRs were located in the same gene body and/or the flanking regions, referred as DMR-associated genes [27]. We identified 9 DMR-associated genes, whose putative promoter region was both hyper- and hypomethylated, whereas the majority of DMRs located in the promoter were either hyper- or hypomethylated (132 and 155 genes, respectively) (Fig. 2D). Further, gene body methylation was observed in 380 genes of which 196 were hypermethylated, 151 hypomethylated, and 33 genes were hyper- and hypomethylated within the gene. In total, 125 genes were identified being differentially methylated downstream the TTS. Here, 55 hypermethylated genes and 60 hypomethylated genes were detected, of which 10 were hyper- and hypomethylated downstream of the TTS. Minimal overlap of hyper- and hypomethylated DMR-associated genes suggests a variant specific methylation pattern.

High differences in methylation level were detected within or in close proximity of genes encoding a beta-galactosidase 5 (DpBGAL5, d_polystachya_ptg000073l_000384.1), a leucine-rich repeat extensin-like protein 4 (DpLRX4, d_polystachya_ptg000080l_000106.1), a shaggy-related protein kinase eta (DpBIN2, d_polystachya_ptg000078l_000124.1), a LOB-domain-containing protein 30 (DpLBD30, d_polystachya_ptg000126l_000709.1), a IQ-DOMAIN 13 protein (DpIQD13, d_polystachya_ptg000004l_000822.1), a TOM1-like protein 9 (DpTOL9, d_polystachya_ptg000005l_000635.1) and a NAC domain-containing protein 35 (DpNAC035, d_polystachya_ptg000011l_000112.1) which were all hypermethylated (see Additional file 3). Among the top hypomethylated genes, an *OCTOPUS* (*DpOPS*, d_polystachya_ptg000164l_000238.1) and a *cinnamoyl-CoA reductase 1* gene (*DpCCR1*, d_polystachya_ptg000085l_000649.1) were detected.

### Enrichment and protein interaction network analysis

**Fig. 3:**
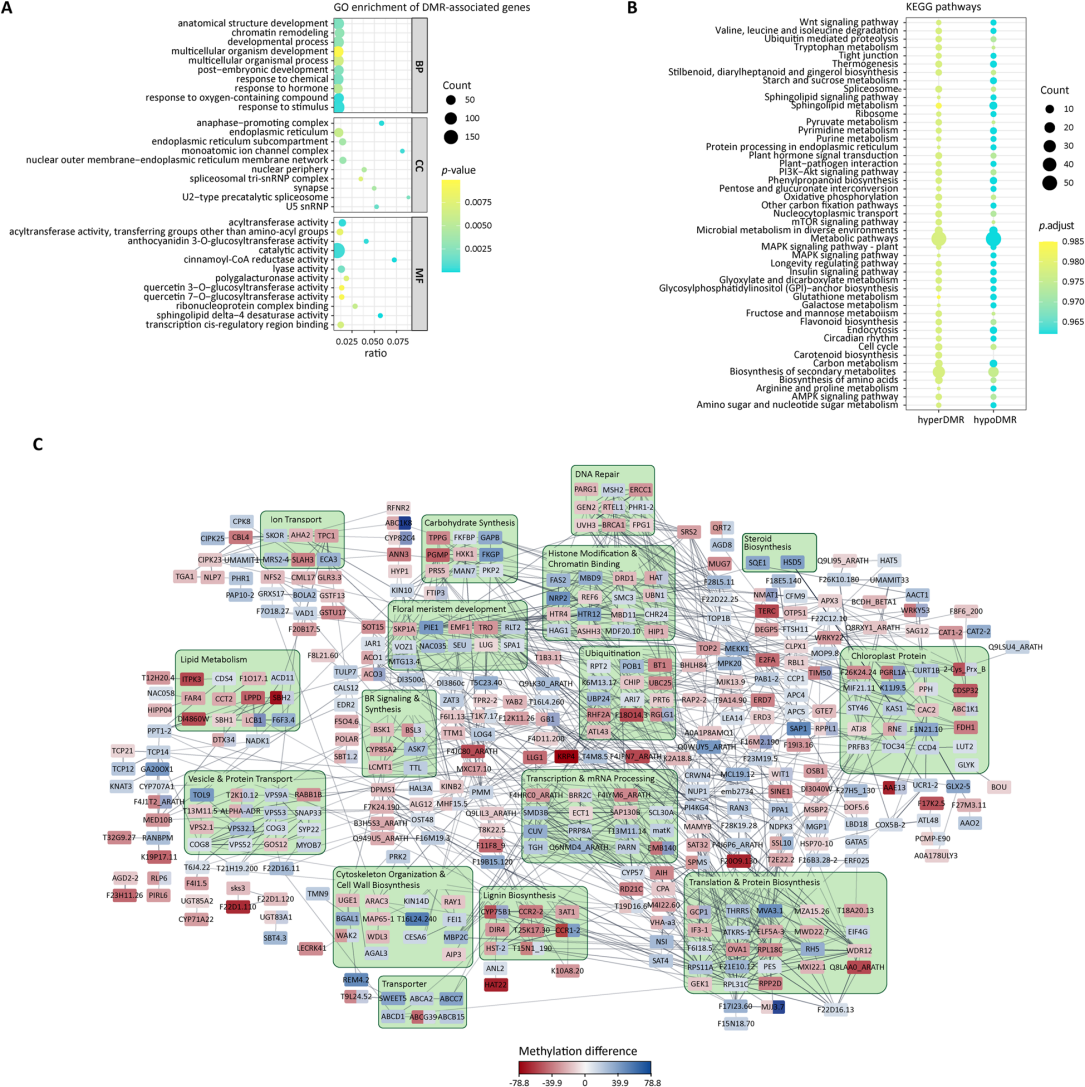
Enrichment and STRING network analysis of DMR-associated genes. **A** GO enrichment analysis of DMR-associated genes (*p*-value < 0.01). ratio: ratio between enriched DMR-associated genes and all genes of a specific GO term. DMR, differentially methylated region; TSS, transcription start site; TTS, transcription termination site; MF, molecular function; CC, cellular component; BP, biological process. **B** Enriched plant-related KEGG pathway of hyperDMR- and hypoDMR-associated genes. **C** Prediction of protein network was performed with DMR-associated genes (methylation difference ≥|10%|, q-value ≤ 0.01) based on protein amino acid sequence. *A. thaliana* was chosen as reference organism in the STRING database. Differences in methylation levels are indicated by the colors blue (hypermethylated, higher methylation level in F2000) and red (hypomethylated, higher methylation level in F60) of the boxes

As DNA methylation is reported to affect gene expression, epigenetic differences might contribute to the phenotypic appearance of the tuber shape var. F60 and F2000. To identify putative candidate genes, Gene Ontology (GO) enrichment analyses were performed. Thereby, 611 of 763 DMR-associated genes (including 4 kb up- and downstream regions) were annotated with at least one GO term. GO enrichment analysis of hyperDMR and hypoDMR-associated genes revealed that most of the enriched GO terms belonged to the domain “biological process” (BP), followed by the domain “molecular function” (MF) and “cellular component” (CC) (Fig. 3A, see Additional file 4). Amongst others, the GO terms “anatomical structure development” (GO: 0048856), response to hormone (GO:0009725), response to light stimulus (GO:0009416), circadian rhythm (GO:0007623) and photomorphogenesis (GO:0009640) were significantly enriched. These terms comprise genes potentially related to cell wall biosynthesis such as *probably cellulose synthase A catalytic subunit 9* (*DpCESA9*, d_polystachya_ptg000004l_000066.1), *expansin-A16* (*DpEXPA16*, d_polystachya_ptg000136l_000378.1) and *LRR receptor-like serine/threonine-protein kinase FEI 1* (*DpFEI1*, d_polystachya_ptg000043l_000014.1). Further, genes encoding putative transcriptional regulators such as the transcription factor CYCLOIDEA (DpCYC, d_polystachya_ptg000147l_001927.1), VOZ1 (DpVOZ1, d_polystachya_ptg000012l_000006.1), TCP14 (DpTCP14, d_polystachya_ptg000042l_001207.1) and a Homeobox-DDT domain protein RLT2 (DpRLT2, d_polystachya_ptg002317l_000003.1) as well as plant hormone biosynthesis genes encoding a putative gibberellin 20 oxidase 1-B (DpGA20ox1B, d_polystachya_ptg000105l_000030.1) as well as a Cytochrome P450 85A1 (DpCYP85A1, d_polystachya_ptg000015l_001384.1), the later potentially involved in brassinosteroid (BR) biosynthesis, were identified belonging to the “anatomical structure development” GO term. Besides the putative *DpCYP85A1* gene, the BR-signaling kinase BSK1 (DpBSK1, d_polystachya_ptg000085l_000115.1) was differentially methylated, which is potentially involved in the BR signal transduction pathway. Additionally, genes encoding for a sphinganine C4-monooxygenase 2 (DpSBH2, d_polystachya_ptg000147l_000925.1, d_polystachya_ptg000042l_000906.1, d_polystachya_ptg000073l_000157.1, d_polystachya_ptg000147l_001222.1), a SUPPRESSOR OF PHYA-105 (DpSPA1, d_polystachya_ptg000078l_000198.1) and a root phototropism protein 2 (DpRPT2, d_polystachya_ptg000002l_000159.1) were identified that were related to photomorphogenesis or response to light stimulus.

To further analyze potential biological pathways that might be affected by the differences of DNA methylation levels, we performed KEGG pathway analyses. However, less than half of the DMR-associated genes (361 of 763 genes) were annotated with KO identifiers (see Additional file 2). Although not significantly enriched, among the plant-related KEGG pathways with the highest number of hyperDMR or hypoDMR-associated genes we identified several genes belonging to the “biosynthesis of secondary metabolites” pathway (ko01110) (Fig. 3B, see Additional file 5). These included also genes of the “phenylpropanoid biosynthesis” pathway (ko00940), in particular, two *DpCCR1* genes (d_polystachya_ptg000085l_000649.1, d_polystachya_ptg000085l_000645.1) that were hypomethylated and potentially involved in lignin biosynthesis [63]. Besides the beforementioned GA and BR biosynthesis genes, two hyperDMR-associated auxin biosynthesis genes (d_polystachya_ptg000015l_000432.1, d_polystachya_ptg000080l_000165.1) encoding an Indole-3-acetaldehyde oxidase (DpAO1) were identified belonging to the secondary metabolite pathway. In total, eight genes putatively involved in plant hormone signal transduction (ko04075) were differentially methylated. These include the beforementioned DpBIN2 and DpBSK1 as well as two hyperDMR-associated *auxin responsive protein saur* genes (DpSAUR, d_polystachya_ptg000150l_000435.1, d_polystachya_ptg000085l_000630.1), the latter potentially related to the auxin signaling pathway. Notably, differentially hypomethylated regions were also identified in genes related to “starch and sucrose metabolism” pathway (ko00500) including the genes *hexokinase-1* (*DpHK1*, d_polystachya_ptg000015l_001205.1) and *phosphoglucomutase* (DpPGM, d_polystachya_ptg000043l_000289.1). Differentially methylated regions were also identified in genes related to transcription and translation. Four genes encoding for proteins of the “spliceosome” (ko03040), which is required for mRNA processing, were shown to be hypermethylated.

In the next step, we analyzed the data for putative interaction and co-regulation networks. Therefore, protein sequences of DMR-associated genes were put into the STRING database [60] and *Arabidopsis thaliana* was chosen as reference organism to assign homologous STRING-IDs for interaction prediction (see Additional file 2). We identified a potential network of genes involved in DNA repair, histone modification, chromatin binding, floral meristem development, transcription, translation, carbohydrate synthesis, lignin biosynthesis, as well as BR signaling and biosynthesis (Fig. 3C). Here, the putative promoter regions of the genes encoding a potential transcriptional corepressor SEUSS (DpSEU, d_polystachya_ptg000010l_000491.1) and a protein PARALOG OF AIPP2 (DpPAIPP2, d_polystachya_ptg000105l_000189.1, STRING name: MTG13.4) were hypermethylated potentially involved in floral meristem development. In *Arabidopsis*, SEU has been described to interact with the transcriptional corepressor LEUNIG (LUG) to regulate meristem activity [64]. Interestingly, a hypoDMR was detected in the gene body of a *DpLUG* gene (d_polystachya_ptg000005l_000530.1) as well, highlighting the potential relation between floral transition and storage organ formation as described in potato [65, 66]. In addition, several of the DMR-associated genes, in particular, *PHOTOPERIOD-INDEPENDENT EARLY FLOWERING 1* (DpPIE1, d_polystachya_ptg000078l_000055.1), *TRAUCO* (DpTRO, d_polystachya_ptg000073l_000699.1) and *methyl-CpG-binding domain-containing protein 9* (DpMBD9, d_polystachya_ptg000136l_000314.1) have been reported to be involved in flowering time [67–70]. Further, we identified the two hypermethylated genes encoding a beta-arabinofuranosyltransferase RAY1 (DpRAY1, d_polystachya_ptg000011l_000495.1) and an LRR receptor-like serine/threonine-protein kinase FEI1 (DpFEI1, d_polystachya_ptg000043l_000014.1), both putatively regulating cell wall biogenesis thereby regulating plant growth [71, 72]. Moreover, additional BR signaling genes were identified including a *serine/threonine-protein phosphatase BSL3* gene (*DpBSL3*, d_polystachya_ptg000125l_000668.1), being hyper- as well as hypomethylated within the gene body, a hyperDMR-associated putative *TITAN-like protein* gene (*DpTTL*, d_polystachya_ptg000173l_000779.1) and a *leucine carboxyl methyltransferase 1 homolog* gene (*DpLCMT1*, d_polystachya_ptg000043l_000402.1) with hypoDMRs located in the putative promoter region.

### Differentially expressed DMR-associated genes

**Fig. 4:**
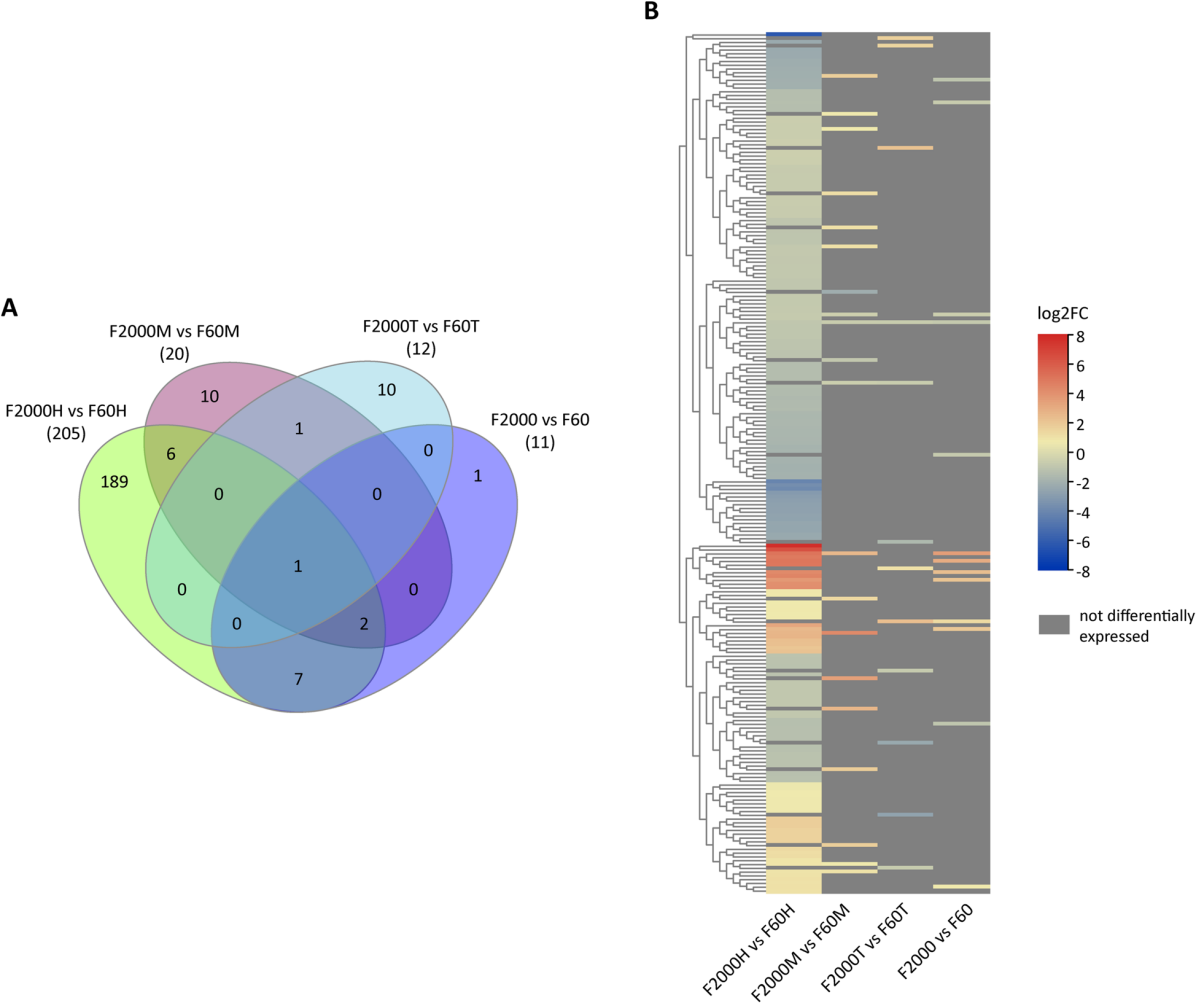
DMR-associated DEG analysis. **A** Venn diagram of DMR-associated genes that were differentially expressed between F2000 and F60 tubers. Transcriptomic data comparison between the head, middle and tip region as well as combined data of the three tuber parts were analyzed (*p*-value ≤ 0.001 and FDR ≤ 0.05). **B** Heatmap of differential expression of DMR-associated genes shown in the Venn diagram. Scales represent differential expression of DMR-associated genes in log2fold-change values. Gray area indicates genes that were not differentially expressed in the specific comparison. H, tuber head region; M, tuber middle region; T, tuber tip region

Next, we investigated the DMR-associated genes of differentially expression using the previously published transcriptomic data of Chinese yam var. F60 and F2000 [40, 46]. In total, 227 DMR-associated differentially expressed genes (DEGs) were identified of which 189 were solely differentially expressed in the upper part of the tubers (head), 10 in the middle part and 10 in the tuber tip (Fig. 4). Combining transcriptomic data of all tuber parts resulted in the identification of 11 DEGs. Interestingly, only one DMR-associated gene was detected to be differentially expressed in all three investigated tuber tissues encoding a potential two pore calcium channel protein 1 (DpTPC1, d_polystachya_ptg000005l_000354.1) showing higher expression in F60 tubers and being hypomethylated within the gene body (see Additional file 6). Inspecting the intersection of DEGs between the different tuber parts, we could identify nine DEGs shared by the tuber head and middle comparisons. Here, four DMR-associated genes were higher expressed in F2000 compared to F60 (d_polystachya_ptg000070l_000583.1, d_polystachya_ptg000078l_000748.1, d_polystachya_ptg000105l_000030.1 and d_polystachya_ptg000239l_000105.1) encoding a putative norbelladine 4'-O-methyltransferase, Beta-galactosidase 2, DpGA20ox1b and a nuclear transcription factor Y subunit B-10, respectively. Besides the *DpTPC1* gene, a gene annotated as *guanylate binding protein* (d_polystachya_ptg000100l_000004.1) was of higher mRNA levels in F60. Interestingly, the investigation of each tuber part individually resulted in the identification of several DMR-associated DEGs related to the floral meristem development including *DpLUG*, *DpSEU*, *DpPIE1*, *DpPAIPP2* and *DpRLT2* being higher expressed in the tuber head of var. F60 compared to F2000, whereas higher transcript levels of *DpNAC035* were detected in F2000. Amongst the top 40 genes of highest methylation level difference, 12 genes were differentially expressed. Besides *DpNAC035*, *DpBGAL5* transcript levels were higher in the upper tuber part, whereas lower in the middle part of F60 compared to F2000. In addition, the plant hormone biosynthesis genes *DpAO1* and *DpGA20ox1B* were lower and higher expressed in the F2000 head, respectively. The latter showed also increased expression in the middle tuber part of F2000. We further identified the two BR signaling DMR-associated genes *DpBSL3* and *DpTTL* being of higher expression in F60 tuber head part. In conclusion, the small number of DMR-associated DEGs indicate the complex relationship between gene expression regulation and DNA methylation and no clear correlation between methylation status and gene expression could be observed.

## Discussion

Epigenome modifications, in particular DNA methylation, have been reported to affect plant organ development and shaping [22, 24, 28]. Spatial and temporal differences in DNA methylation have been observed between various types of tissues as well as throughout different developmental stages suggesting an important role during plant specific processes including the regulation of plant architecture, fruit development and ripening, storage organ morphology, and plant response to biotic and abiotic stresses [7, 8, 11, 12, 15, 23, 73–75].

In the current study, we conducted whole-genome bisulfite sequencing of two tuber shape variants F2000 (short, thick) and F60 (long, thin) and performed differential methylation analysis in CG, CHG and CHH context to investigate epigenetic effects on tuber morphology in Chinese yam. Highest number of methylated cytosines were observed in CG context, followed by CHG and CHH. Although DNA methylation pattern can vary across plant species, CHH methylation has been reported to universally be the lowest while CG methylation was shown to be the predominant type, thus our data are in agreement with results of other angiosperm species [76]. The overall observed low CHH methylation level coincides with other mainly asexual propagated plant species, in which an association between progressively irreversible loss of CHH methylation and clonal propagation has been speculated [76, 77]. Further, it has been speculated that DNA methylation might play a role in plant adaptation processes in which each methylation context potentially plays different temporal roles [78]. Changes of DNA methylation in CHH context was hypothesized to be a rather short-term response to environmental cues whereas CG methylation could act in a more long-term way [78]. This assumption and the lower correlation in CHH context between the biological replicates could be an indication of a minor role of CHH methylation in Chinese yam tuber development, and identified DMRs in this context should be treated with caution.

As TSS region is typically enriched in transcription factor binding sites, the observed decreased methylation level around the TSS as well as the TTS in the three contexts could be due to transcriptional repression avoidance as proposed for other plant species [27, 79]. The comparable methylation pattern observed in both Chinese yam variants to previous reports in angiosperms suggests a similar regulatory function of DNA methylation on gene transcription. Examination of methylation level differences between both variants revealed that more than one DMR was associated with a single gene and analysis of intersections disclosed 52 DMR-associated genes that were hyper- and hypomethylated within the respective genetic feature. Although the usage of genome of variant F60 as reference for mapping may cause bias in DMR calling, which cannot be ruled out at the current time, these observations, especially the small number of genes that were hyper- and hypomethylated, supports the assumption that DNA methylation variation might contribute to differences in storage organ morphology.

### Role of circadian rhythm and photoperiodism in tuberization onset

In both of our omic data sets, we identified genes of the same biosynthetic or signaling pathways including components potentially involved in regulating circadian rhythm, photoperiodism, plant hormone biosynthesis and signaling and cell wall biosynthesis [40] indicating a potential synergistic relationship between DNA methylation and gene expression in tuber development as described for other plant species [73, 74]. For instance, genes encoding DpPIE1, DpTRO and DpMBD9, whose homologs have been reported to be involved in histone modification and thereby regulating *AtFLC* expression in *Arabidopsis* [67–70]*,* were differentially methylated between F60 and F2000. Studies have shown that AtFLC represses the transcription of *FLOWERING LOCUS T* (*AtFT*) resulting in delayed flowering [80]. However, we could neither identify any ortholog of FLC in the transcriptomic data nor in the genomic data. Similar, no *FLC* homolog has been identified in rice [81, 82]. Nevertheless, DpPIE1 and DpRLT2 might as well control the chromatin structure in Chinese yam thereby resulting in differences the transcript levels of downstream target genes which could be related to tuber development and needs to be further investigated in future research.

We further identified a hypermethylated *DpVOZ1* and *DpSPA1* gene in Chinese yam as another potential component of the circadian rhythm pathway. Based on findings in *Arabidopsis* mutants *atvoz1-1* and *atvoz1-2*, transcription of *AtFT* was proposed to be activated by interaction of AtVOZ1 and CONSTANS (AtCO) to promote flowering [83, 84]. In addition, AtSPA1-AtCO interaction has been suggested to negatively regulate *AtFT* expression controlling photoperiodic flowering [85]. Previous studies have suggested an important role of components of the circadian clock in the tuberization in potato [86, 87]. One major key player is the tuberigen SELF PRUNING 6 A (StSP6A), a FT homolog, that acts as a mobile signal inducing tuberization [88–90]. Its expression is negatively controlled by StCO, a CONSTANS homolog, that directly induces the expression of *StSP5G*, which in turn represses *StSP6A* [65, 86]. Several transcription factors, including DpCYC and DpTCP14, showed differential methylation. In potato, the TCP transcription factor BRANCHED1 (StBRC1b) represses tuberization in aerial buds via its interaction with StSP6A [91]. Thus, DNA methylation could affect the interplay of DpVOZ1, DpSPA1, DpCYC and DpTCP14 regulating *DpFT* expression or protein activity and eventually controlling tuber development. Only recently, the FT homolog DpFT3a was identified as potential tuberigen in Chinese yam and its overexpression in transgenic potato plants resulted in a severe tuberizing phenotype [46]. Different methylated genes related to the photoperiodic-tuberization pathway were also identified in the comparisons between photoperiod-insensitive and photoperiod-sensitive potato genotypes indicating a role of epigenetics in tuberization further supporting our assumption [92].

In addition, it is striking that a *DpSEU* and *DpLUG* gene were both differentially methylated and of lower expression in F2000 head tuber part compared to F60. The interaction of AtSEU and AtLUG has been shown to repress microRNA *172* (*miR172*) expression to control floral organ development [64, 93, 94]. Like in *Arabidopsis*, *miR172* expression promote flowering in potato but also accelerates tuberization [95]. Further, decreased endogenous levels of GA were reported to promote swelling whereas high levels favor elongation in the potato stolon tip [96]. Hence, differential DNA methylation of genes related to the circadian clock as well as GA biosynthesis pathway (*DpGA20ox1B*) might be associated with the onset of tuberization in Chinese yam which could result in different time points of tuber initiation between F60 and F2000, thus potentially affecting biomass production.

### Differential methylation of BR-related genes supports its function in tuber shape

Brassinosteroids play an important role in plant development and organ growth regulating e.g. cell division, cell elongation, and photomorphogenesis [97–99]. Genes related to BR biosynthesis or signaling have been proposed to be candidates for crop improvements with emphasis on biomass production [100]. Moreover, BRs are suggested to be involved in tuberization as knockdown of the BR receptor *BR-INSENSITIVE1* (*StBRI1*) resulted in reduced plant growth and reduction in tuber weight and yield in potato [101]. A role of BRs in organ shape was demonstrated by the knockout of *globe*, which putatively encodes a BR hydroxylase, resulting in a globe phenotype in tomato [102]. Recently, we identified differentially expressed BR synthesis and signaling as well as putative BR-regulated genes in the comparisons between Chinese yam var. F60 and F2000 [40]. The higher BR levels in the tuber tip of var. F2000 and increased tuber width-to-length ratio after exogeneous 24-epi-brassinolide application suggests a function of BRs in Chinese yam tuber development [40]. In our methylome comparison, we identified DMR-associated genes related to BR biosynthesis (*DpCYP85A1*) as well as BR signaling (*DpBSL3, DpBIN2, DpBSK1, DpLCMT1, DpTTL*). Recently, WGBS analysis of *D. zingiberensis* revealed a significant difference of methylation level of BR-related genes compared to overall methylation pattern of coding genes [103]. In the same study, it was demonstrated that BR treatment directly affected DNA methylation level including BR pathway genes. Thus, alteration in DNA methylation of these pathway genes could be an explanation for the detected differences in BR content and ultimately regulating downstream target genes causing the Chinese yam tuber shape variation. Networks between organ shape-related and BR-related genes were previously described [104–108]. Amongst others, interactions between OsGSK2, a BIN2 homolog, and proteins of the OVATE family (OsOFPs) were confirmed that regulate BR signaling and grain shape [105, 106, 108]. Proteins of the OVATE family (OFPs) have been demonstrated to regulate storage organ shape in several plant species such as radish and potato, in which overexpression resulted in round and suppression in elongated shapes probably by controlling cell division [109, 110]. Interestingly, premature ectopic expression of *SlLA* (*TCP4*/*LANCEOLATE*) in the semi-dominant *La* mutant resulted in repression of *OVATE* and elongated tomato fruits [111]. The observed higher expression of *DpOFP* genes in F2000 tubers compared to F60 [40] may be regulated through an interplay of transcriptional regulation by DpTCPs and the BR signaling pathway. Another potential candidate regulating Chinese yam tuber shape is the DMR-associated *DpIQD13* gene. OsGSK2 was shown to interact with OsGSE5, an IQ67 domain (IQD) protein negatively regulating grain width, which in turn controls BR-responsive genes [112]. Similar to observation in other plant species, where IQD proteins have been reported to regulate organ shape through microtubule organization [113–115], we hypothesize that DpIQD may play a comparable role in regulating tuber width in Chinese yam. Regulation of BR signaling could be further fine-tuned by DpOPS, which was differentially methylated. In cabbage (*Brassica rapa* L. spp. *Pekinensis*), BrOPS was shown to sequester BrBIN2 to the plasma membrane via protein–protein interaction, thereby positively regulating BR signaling and controlling head shape [116]. BRs are also known to control cell wall composition by regulating the expression of primary and secondary cell wall biosynthesis genes thereby controlling cell growth [117]. The identified cell wall biosynthesis genes in our analysis might be regulated by DNA methylation and/or the BR pathway. In *Populus*, comparison of juvenile and mature wood revealed DMR-associated DEGs of the BR signaling and response and cell wall biogenesis pathway, and it has been hypothesized that DNA methylation affected gene expression and wood properties [118]. We also speculate that differential methylation of these hormone biosynthesis and signaling pathway genes might be associated with transcriptional alteration of downstream target genes. Although gene body methylation has been reported to result in higher transcript levels, there is also some evidence of a suppressing function on gene transcription as well as a regulatory function on mRNA splicing efficiency to control alternative transcript production [16–18, 20]. However, similar to observations in other plant species [27, 73, 74, 92, 119], only a confined number of differentially expressed DMR-associated genes were identified. It has been concluded that there exists a complicated relationship between DNA methylation and gene expression and that genetic, epigenetic and transcriptomic evolutionary changes might collaboratively influence plant traits [73, 74]. In our study, we selected the enlargement stage for investigation, as changes in Chinese yam tuber width and length become most apparent during this phase [120]. Therefore, transcriptomic as well as epigenetic data, limited to a specific developmental stage, may not fully capture the dynamic gene expression and methylation patterns in Chinese yam, particularly for genes with circadian rhythm. While providing valuable insights, the dynamic interplay between DNA methylation and gene expression in Chinese yam may not be fully represented. Moreover, DNA methylation might regulate alternative splicing events resulting in transcript diversity, which needs to be further investigated. Further research on how DNA methylation directly or indirectly affects gene expression by incorporating time-series transcriptomics may help to further elucidate the role of epigenetics in tuber shaping in Chinese yam.

## Conclusion

To our knowledge this is the first report on the potential role of epigenetics in Chinese yam tubers. We identified several putative DMR-associated genes that might be associated with tuber shape in a complex regulatory network. Our findings provide a foundation for future research aimed at understanding the epigenetic basis of tuber development in Chinese yam and identifying candidate genes for breeding programs.

## Supplementary Information

Additional file 1: Statistics of genome-wide bisulfite sequencing data

Additional file 2: Matrix of differentially methylated regions between Chinese yam var. F60 and F2000

Additional file 3: Top 20 hyperDMR and hypoDMR-associated genes

Additional file 4: Enriched GO terms of DMR-associated genes

Additional file 5: Enriched KEGG pathways of DMR-associated genes

Additional file 6: DMR-associated differentially expressed genes

## Data Availability

The whole-genome bisulfite sequencing data of this article are available at the National Center for Biotechnology Information (NCBI) SRA database under the BioProject ID: PRJNA1336429 (https://www.ncbi.nlm.nih.gov/sra/PRJNA1336429). Previously published genome data of *D. polystachya* variant F60 [46] that was used during the current study is available at the European Nucleotide Archive (ENA) under the project: PRJEB82723 (https://www.ebi.ac.uk/ena/browser/view/PRJEB82723). Previously published tuber transcriptomic data of *D. polystachya* variant F60 and F2000 [40] used in the current study are available at the NCBI SRA database under the BioProject ID: PRJNA942579 (https://www.ncbi.nlm.nih.gov/ bioproject/PRJNA942579).
